# Beverages Consumption and Oral Health in the Aging Population: A Systematic Review

**DOI:** 10.3389/fnut.2021.762383

**Published:** 2021-10-27

**Authors:** Roberta Zupo, Fabio Castellana, Sara De Nucci, Vittorio Dibello, Madia Lozupone, Gianluigi Giannelli, Giovanni De Pergola, Francesco Panza, Rodolfo Sardone, Heiner Boeing

**Affiliations:** ^1^Unit of Data Sciences and Technology Innovation for Population Health, National Institute of Gastroenterology “Saverio de Bellis,” Research Hospital, Bari, Italy; ^2^Department of Orofacial Pain and Dysfunction, Academic Centre for Dentistry Amsterdam (ACTA), University of Amsterdam and Vrije Universiteit Amsterdam, Amsterdam, Netherlands; ^3^Scientific Direction, National Institute of Gastroenterology “Saverio de Bellis,” Research Hospital, Bari, Italy; ^4^Unit of Geriatrics and Internal Medicine, National Institute of Gastroenterology “Saverio de Bellis,” Research Hospital, Bari, Italy; ^5^Department of Epidemiology, German Institute of Human Nutrition Potsdam-Rehbrücke, Nuthetal, Germany

**Keywords:** drinks, beverages, oral health, oral frailty, aging, older people, systematic review

## Abstract

Little study has yet been made of the effect of different beverages on oral health outcomes in the aging population. The purpose of this systematic review is to evaluate the association between different beverages, including alcohol intake, coffee, milk, tea, and sugary drinks, and a cluster of oral health outcomes, including periodontal disease, oral dysbiosis, and tooth loss in older adults. The literature was screened from the inception up to May 2021 using six different electronic databases. Two independent researchers assessed the eligibility of 1308 retrieved articles regarding inclusion criteria; only 12 fitted the eligibility requirements, representing 16 beverage entries. A minimum age of 60 was the inclusion criterion. No exclusion criteria were applied to outcomes assessment tools, recruiting facilities (hospital or community), general health status, country, and study type (longitudinal or cross-sectional). The consumption of alcoholic beverages was expressed as alcohol intake in all eligible studies, thereby replacing alcoholic beverages in the analysis. The quality of evidence was judged as moderate for alcohol and low or very low for beverages. In regard to oral health in the elderly, the review identified information on alcohol (56.25%), followed by coffee (18.75%), milk (12.50%), tea (6.25%), and sugary drinks (6.25%). Alcohol, sugary drinks, and coffee were found to be related to tooth loss. Periodontal disease was inversely related to coffee and milk, but fostered by alcohol consumption. In one article, tea but not coffee seemed to improve oral microbiota. In summary, alcohol seems to be a driver for tooth loss and periodontal disease in the aging population. However, more research is needed to gain a more solid knowledge in this research area.

**Systematic Review Registration:** https://www.crd.york.ac.uk/prospero/, PROSPERO, Identifier: CRD42021256386.

## Introduction

Healthy aging is critical to a good quality of life in older adults, and to reducing the healthcare system burden. In this context, the deterioration of oral function, most often combined with an altered perception of taste and thirst, is a well-known but often overlooked adverse feature of the aging process (1). Tracing back along the etiopathogenic trajectories of age-related oral deterioration, the evidence suggests that a pattern consisting of poor dentition, tooth decay, altered microbiota, and periodontal disease delineates a causal path linked to immune and cellular senescence and subsequently to physical and cognitive deterioration (2, 3). Recently, concerns about poor oral health are in the spotlight when considering the aging population, especially those frail. Oral health represents an essential aspect of health, life satisfaction, quality of life, and self-perception, and this feature in turn may indirectly affect aging trajectories through biological interaction paths with several functional domains (4).

There is further evidence for lifestyle components associated with an acceleration of poor oral health, where diet is a key factor (5). Diet could be seen to contribute to the etiology of poor oral health, also as a consequence of a dysfunctional oral health pattern, particularly in older adults. This can lead to feeding difficulties, resulting in changes in food choices likely to predispose to an increased risk of malnutrition and sarcopenia, resulting in loss of muscle mass, muscle strength, and physical performance (6–9).

Dietary intake of all types of fluids, including water and various beverages such as tea, coffee, milk, sugary drinks, fruit juices, and alcoholic drinks, serves to prevent dehydration, another possible problem of aging due to an altered thirst perception (1, 10). This generally positive role of the intake of fluids could be counterbalanced by adverse effects caused by the mixed composition of popular beverages. These affect oral health, inducing problems such as dental caries and erosion. In this context, sugary beverages have been identified as drivers of the risk, showing a clear dose-response relationship (11). Alcohol has also been extensively explored, and a recent meta-analysis of observational studies has suggested excessive consumption to be associated with a higher incidence of periodontitis, thus advocating the inclusion of this dietary item as a noteworthy yardstick in periodontal risk management (12). Against this preventive backdrop, the aging population comes to the forefront again if considering that heavy alcohol consumption is more prevalent among middle-aged and older people compared to the youngest (13).

The level of exposure to beverage consumption, known as a potentially modifiable lifestyle factor, and the potential interaction with oral health outcomes so far refereed by scientific evidence leaves an exploratory window for conceptual synthesis, useful in terms of risk management. The potential interaction between nutrition and oral health prompted us to systematically evaluate the literature on the association between exposure to different beverages and poor oral health outcomes in the aging population.

## Methods

### Search Strategy and Data Extraction

The present systematic review followed the Preferred Reporting Items for Systematic reviews and Meta-Analyses (PRISMA) guidelines, adhering to the PRISMA 27-item checklist (14). An *a priori* protocol for the search strategy and inclusion criteria was established and registered, without particular amendments to the information provided at registration, on PROSPERO, a prospective international register of systematic reviews (CRD42021256386). We performed separate searches in the US National Library of Medicine (PubMed), Medical Literature Analysis and Retrieval System Online (MEDLINE), EMBASE, Scopus, Ovid, and Google Scholar databases to find original articles inquiring into any association between the exposure to different beverages and oral health outcome(s). Thus, the main goal was to evaluate the association between exposure to different beverages and poor oral health outcomes in the aging population. We also considered the gray literature using the largest archive of preprints https://arxiv.org/ in the study selection phase, and http://www.opengrey.eu/ database to access conference remarkable conference abstract and other not peer reviewed material. Since we chose only observational studies to be included, the search strategy followed PECO (Populations, Exposure, Comparator, and Outcomes) concepts (15), including populations (60+ years of age), exposure (alcohol and beverages such as milk, tea, coffee, and sugar-sweetened drinks), comparators (exposure levels), and outcomes (oral health outcomes). Exposure factors were selected to include major groups of beverages, i.e., alcoholic beverages (as alcohol intake), coffee, milk, tea, and sugary drinks, regardless of the assessment tool(s) employed. Outcomes included all types of deteriorating oral health conditions, i.e., periodontal disease, oral dysbiosis (gingivitis), and tooth loss. It should be borne in mind that in articles regarding oral health, the consumption of alcoholic beverages is usually calculated as alcohol intake [except for a single report that also looks at alcoholic beverages (16)], so this was the exposure taken into account in this study.

The search strategy used in PubMed and MEDLINE and adapted to the other four electronic sources is shown in detail in Supplementary Table 1. In the literature search, no time limit was set and articles were retrieved until June 1st, 2021. No language limitation was introduced. Two researchers (RZ, VD) searched the papers, reviewed titles and abstracts of articles retrieved separately and in duplicate, checked full texts, and selected the articles for inclusion in the study. Technical reports, letters to the editor, and systematic and narrative review articles were excluded. Inter-rater reliability (IRR) was used to estimate inter-coder agreement, and then the κ statistic as a measure of accuracy and precision. A coefficient k of at least 0.9 was obtained in all data extraction steps, both according to PRISMA concepts and along with the quality assessment steps (17).

### Inclusion Criteria, Data Extraction, and Registration

Exposure and outcome had to be referred to a population aged 60 years or older. No criterion was applied to the recruitment settings (hospital or community) or health status of the study population (general population or groups with specific characteristics). Potentially eligible articles were identified by reading the abstract and, if eligible, reading the full-text version of the articles. For each article selected, the best statistical approach in respect to confounding as applied in evaluating the magnitude of the effect size for associations was considered. Data were cross-checked, any discrepancies were discussed, and disagreements were resolved by a third investigator (RS).

The following information was extracted by two investigators (RZ, VD) separately and in duplicate in a piloted form: (1) general information about single studies (author, year of publication, country, settings, design, sample size, age); (2) type of beverage (including alcohol) exposure; (3) outcome(s) regarding oral health conditions, including periodontal disease, oral dysbiosis, and tooth loss; (4) type of oral health outcome assessment tool(s); (5) main finding(s); (6) effect size of the association between exposure and outcome.

All references selected for retrieval from the databases were managed with the MS Excel software platform for data collection. Lastly, data extracted from selected studies and stored in the database were structured as tables of evidence.

### Quality Assessment Within and Across Studies and Overall Quality Assessment

The methodological quality of the included studies was independently appraised by paired investigators (VD, FC), using the National Institutes of Health Quality Assessment Toolkits for Observational Cohort and Cross-Sectional Studies (18, 19). The ratings: high (good), moderate (fair), or poor were assigned to studies according to the criteria stated in the toolkit. This tool contains 14 questions that assess several aspects associated with the risk of bias, type I and type II errors, transparency, and confounding factors, i.e., study question, population, participation rate, inclusion criteria, sample size justification, time of measurement of exposure/outcomes, time frame, levels of the exposure, defined exposure, blinded assessors, repeated exposure, defined outcomes, loss to follow-up, and confounding factors. Items 6, 7, and 13 do not refer to cross-sectional studies, and the maximum possible scores for cross-sectional and prospective studies were 8 and 14, respectively. Disagreements regarding the methodological quality of the included studies between the two investigators were resolved through discussion until a consensus was reached together with a third investigator (RS). A modified version of the Grading of Recommendations Assessment, Development, and Evaluation (GRADE) rating system was used to assess the overall quality of evidence of the studies included in the present systematic review (16, 20). The following factors were considered: the strength of association for beverage consumption and related oral health outcomes, methodological quality/design of the studies, consistency, directedness, precision, size, and (where possible) dose-response gradient of the estimates of effects across the evidence base. Evidence was graded as very low, low, moderate, and high, as in the GRADE rating system.

## Results

**Figure 1:**
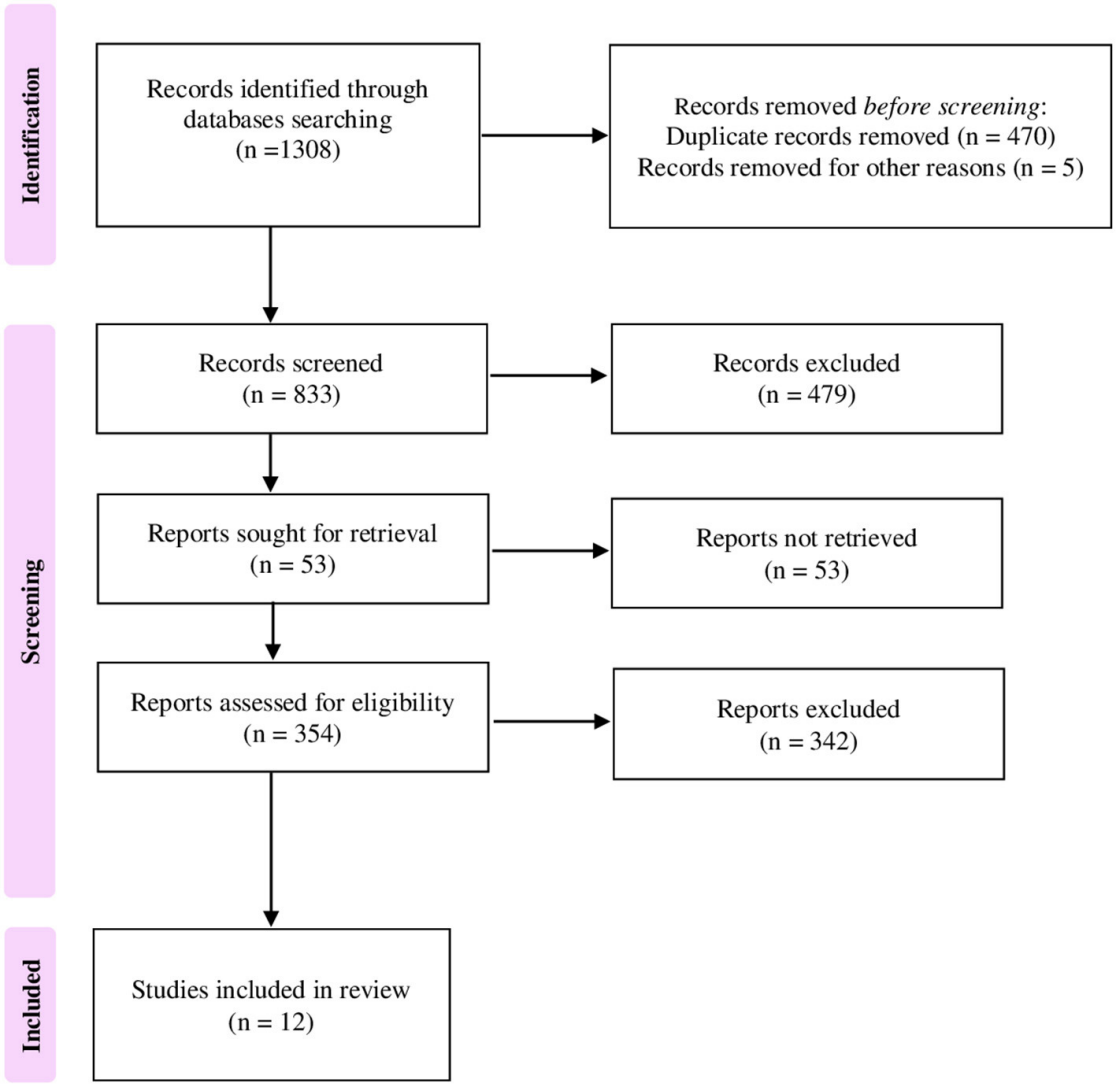
Preferred Reporting Items for Systematic Reviews and Meta-analyses (PRISMA) flow chart illustrating the number of studies at each stage of the review.

**Figure 2:**
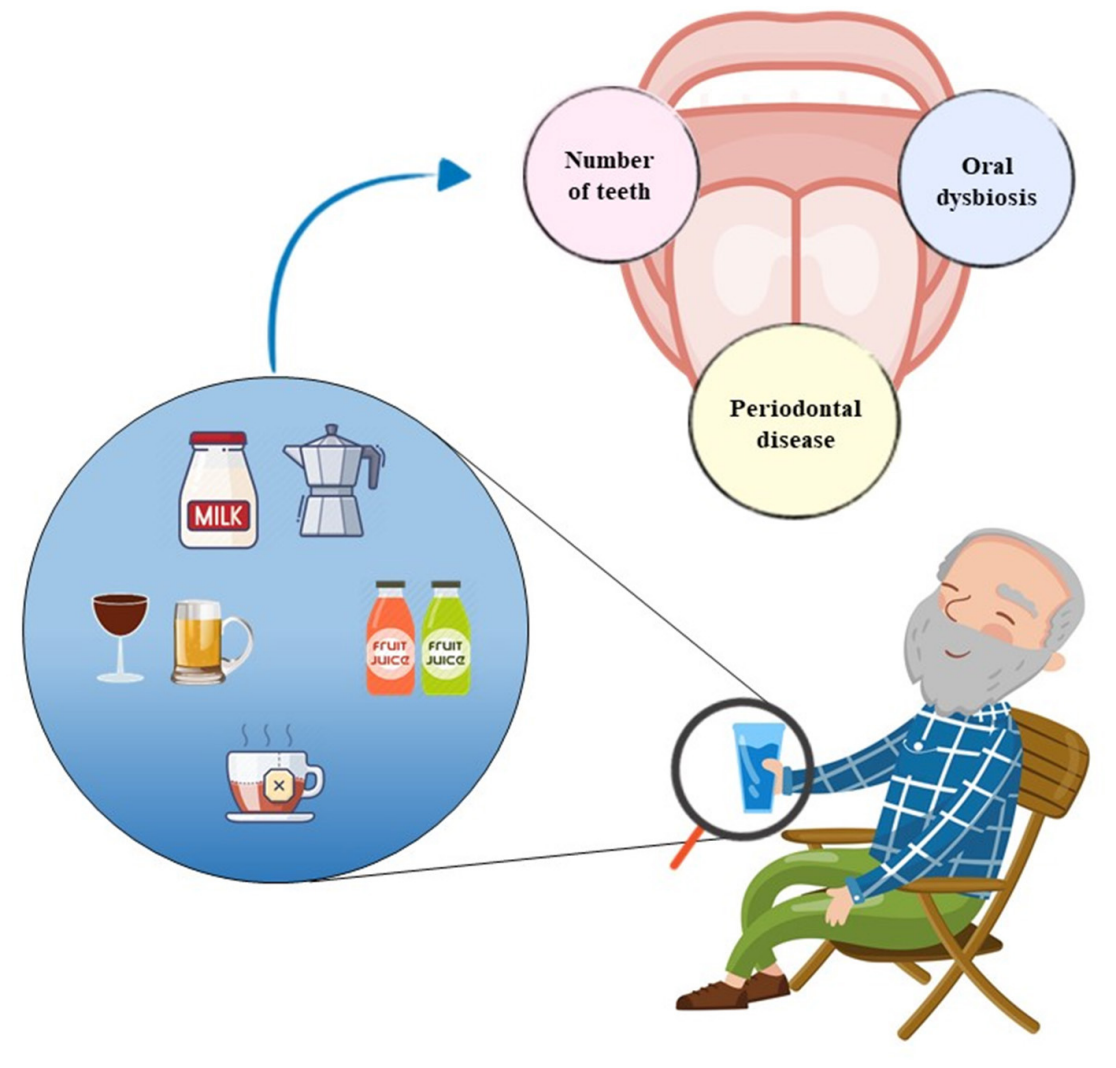
Graphic overview of the findings.

The preliminary systematic search of the literature yielded 1,308 records. After excluding duplicates, 833 were considered potentially relevant and retained for the analysis of titles and abstracts. Then, 479 were excluded for not meeting the characteristics of the approach or the review goal. After reviewing the full text of the remaining 354 records, only 12 met the inclusion criterion of age and were included in the final qualitative analysis (21–32). The Preferred Reporting Items for Systematic Reviews and Meta-analyses (PRISMA) flow chart illustrating the number of studies at each stage of the review is shown in Figure 1. The final study base included 12 articles reporting on four different beverages, i.e., coffee, milk, tea, and sugary drinks, as well as alcohol intake. Figure 2 shows a graphic overview of the findings.

Details of the design (cohort or cross-sectional), sample size (*N*) and sex ratio (%), minimum or and mean (SD) age or age range, setting (community or hospital), and country of individual studies are shown in Table 1. The cross-sectional design (75%, *N* = 9) predominated over the longitudinal design (25%, *N* = 3). Recruitment settings were mostly community-based (91.7%; 11 out of 12), but also included one hospital-based study. The geographic distribution of the studies extended over Asia, Europe, and America (*N* = 4 of 12 each, so 33.3% per continent). In accordance with the inclusion criterion, subjects were aged over 60 and were predominantly 65+ years. Among all 12,223 subjects of the studies, gender was balanced (approximately 50% each). Most studies focusing on alcohol intake found a positive association with tooth loss (3 out of 5 selected studies) (16–19, 25) and periodontal disease (3 out of 4 selected studies) (20, 22, 23, 26). Overall, alcohol intake was the most represented item of the findings (56.25%, *N* = 9 of 16), followed by coffee (18.75%, *N* = 3 of 16), milk (12.50%, *N* = 2 of 16), tea (6.25%, *N*= 1 of 16), and sugary drinks (6.25%, *N*= 1 of 16).

**Table 1:** Selected studies investigating beverage consumption and oral health status in older age (*N* = 12).

**Author, Year (Ref.)**	**Beverage**	**Oral Health Item**	**Oral Health Assessment Tool**	**Design (follow-up)**	**Setting**	***N***	**Age**	**Sex**	**Country**	**Findings**
Drake et al. (1995) (21)	Alcohol	Number of teeth	Interview (questionnaire)	Longitudinal, 3-year	Community	810	65+	ND	America (USA)	Increased alcohol consumption was associated with tooth loss in Whites
Norlén et al. (1996) (22)	Coffee Alcohol	Number of teeth	Interview (questionnaire)	Cross-sectional	Community	483	68	100% M	Europe (Sweden)	High consumption of coffee or alcohol were associated with fewer remaining teeth
Hanioka et al. (2007) (23)	Alcohol	Number of teeth	Interview (questionnaire)	Cross-sectional	Community	6,805	70+	39% M, 61%F	Asia (Japan)	The prevalence of tooth loss was lower in current drinkers and the relationship approached significant levels in females
Yoshihara et al. (2009) (24)	Milk alcohol	Periodontal disease	Root caries and periodontal disease. Evaluation of the clinical attachment level (CAL)	Longitudinal, 6-year	Community	600	70	51% M, 49% F	Asia (Japan)	There was a significant negative relationship between the amount of daily milk intake and the number of root caries. The number of root caries events during the 6 years was significantly lower among subjects with a greater intake of milk products. Alcohol consumption was positively associated with the number of periodontal disease events during the same time period
Heegaard et al. (2011) (25)	Alcohol	Number of teeth	Number of remaining teeth, including third molars, dichotomized as ≥20 vs. <20 remaining teeth	Cross-sectional	Community	783	65+ (65–95)	ND	Europe (Denmark)	Alcohol consumption, wine drinking, and wine and spirits preference among women were associated with a higher number of teeth compared with abstainers
Adegboye et al. (2012) (26)	Milk	Periodontal disease	Number of teeth with attachment loss ≥3 mm	Cross-sectional	Community	135	65+	47% M, 53% F	Europe (Denmark)	Dairy calcium, particularly from milk and fermented products, may protect against periodontitis
Machida et al. (2014) (27)	Coffee Alcohol	Periodontal disease	Probing pocket depth (PPD) and clinical attachment level (CAL), bleeding on probing (BOP), plaque levels evaluation	Cross-sectional	Hospital	414	66.4 ± 9.9	20.8% M, 79.2% F	Asia (Japan)	There appears to be an inverse association between coffee consumption (≥1 cup/day) and the prevalence of severe periodontitis
Hach et al. (2015) (28)	Alcohol	Periodontal disease	Pocket depths, clinical attachment loss, distance from the enamel–cementum junction to the bottom of the periodontal pocket evaluation	Longitudinal, 20-year	Community	168	65+	45.8% M, 54.2% F	Europe (Denmark)	Early consumption of alcohol may increase the odds of having periodontitis 20 years later. The results of long-term alcohol consumption, from 1981 to 2003, and periodontitis showed that heavy drinkers tended to have a higher odds ratio for periodontitis compared to light drinkers
Tiwari et al. (2016) (29)	Sweet beverages	Number of teeth	Interview (questionnaire)	Cross-sectional	Community	308	65+	47.3 M, 52.7% F	America (USA)	Tooth loss was significantly associated with the consumption of one or more than one sweet beverage per day
Laguzzi et al. (2015) (30)	Alcohol	Number of teeth	Interview (questionnaire)	Cross-sectional	Community	341	65+	63% M, 37% F	America (Uruguay)	Lack of functional dentition, severe tooth loss, and edentulism were found to be associated with frequent consumption of alcohol
Suwama et al. (2018) (31)	Alcohol	Periodontal disease	Probing pocket depth (PPD) and clinical attachment level (CAL)	Cross-sectional	Community	438	73	53.8% M, 46.2% F	Asia (Japan)	An increased mean CAL was significantly associated with heavy alcohol drinking in community-dwelling elderly Japanese
Peters et al. (2018) (32)	Coffee Tea	Oral microbiota	16S rRNA gene sequencing	Cross-sectional	Community	938	65+	ND	America (USA)	Higher tea intake was associated with greater oral microbiota richness and diversity, and shifts in overall community composition. Coffee was not associated with these microbiome parameters

*ND, not defined; N, number of study participants; M, males; F, females; CAL, clinical attachment level; PPD, probing pocket depth; BOP, bleeding on probing*.

Regarding the measurements, alcohol intake was reported as grams per day or week or per body weight of the enrolled subjects. Tea, coffee, milk, and sugary beverages were quantified as daily cups. The three outcomes recorded in the studies referred to tooth loss, periodontal disease, and oral dysbiosis. The assessment method for tooth loss was consistent across the studies, detected either by questionnaire or clinical assessment, whereas oral dysbiosis was estimated by 16S rRNA gene sequencing. Conversely, assessment measures for periodontal disease included pocket depth on probing (PPD), clinical attachment level (CAL), bleeding on probing (BOP), or assessment of the distance from the enamel-cement junction to the bottom of the periodontal pocket.

Of the studies investigating alcohol, 5 of 9 investigated tooth loss as the oral outcome (21–23, 25, 30), while the remaining four investigated periodontal disease (24, 27, 28, 31). Three of five studies of alcohol and tooth loss found a negative relation, while the other two studies found the opposite, so a beneficial relation (23, 25). A single study has extended the analysis also to specific alcoholic beverage consumption, concluding that more than six glasses of wine (in females) and beer (in males) were associated with lower odds of having fewer teeth (16), confirming their results on alcohol. The studies about alcohol and periodontal disease showed an inverse relation (24, 27, 28, 31), except for a single study (22).

Regarding beverages, the conclusion was hampered by the small number of studies. Coffee and milk were found to be inversely related to periodontal disease (24, 26, 27), while sugary beverages and coffee were associated with tooth loss (22, 29). Tea but not coffee appeared to improve oral microbiota, although only one study has been recorded to date (32).

We found a moderate (*N* = 9) to low or (*N* = 3) very low (*N* = 4) methodological quality of the studies (Table 2). An overview of quality ratings within (panel A, Supplementary Material) and across studies (panel B, Supplementary Material) is provided in Table 2, highlighting areas with higher or lower ratings. Bias was seen primarily in the domains of sample size justification (selection bias) and blinded assessors (detection bias) (100%, 12/12 studies for both domains), and to a lesser extent in the domains of the participation rate (3/12 studies, 25% of studies with a higher risk of bias), different levels of exposure (2/12 studies, 17% of studies with a higher risk of bias), outcome measurement (3/12 studies, 25% of studies with a higher risk of bias), and statistical correction for confounding factors (3/12 studies, 25% of studies with a higher risk of bias) (Table 2, panel B, Supplementary Material).

**Table 2:** Summary of findings about different beverages associated to oral health items in older age.

**Exposure**	**Evidence base**	**Strength of association**	**Strength of evidence (GRADE)**
Alcohol	Nine studies	Number of alcoholic drinks/week and loss of at least one tooth in 3 years: logistic regression estimate of 0.483 (OR 1.62), significant	⊕⊕⊕ Moderate
	*n* = 10,842	(21)	
		Number of teeth within three categories of increasing alcohol consumption. Less teeth were observed with increasing alcohol intake. ANOVA of lowest (0–110 g alcohol/week) and highest (>250 g/week) category was significant *p* <0.05	
		(22)	
		Males: Tooth loss with current alcohol consumption (>20 g of alcohol/day for 3 or more days/week) vs. never: OR 0.71, 95% CI 0.47–1.09. Females: Tooth loss with current alcohol consumption (>20 g of alcohol/day for 3 or more days/week vs. never): OR 0.25, 95% CI 0.07–0.84	
		(23)	
		Regression analysis between alcohol consumption (g/kg) and periodontal disease events over 6 years: Positive regression coefficient of 1.87, 95% CI 0.08–3.66, *p* = 0.041	
		(24)	
		OR for having a low number of teeth (>20) with moderate or heavy alcohol drinking vs. abstainers: females OR 0.40, 95% CI 0.22–0.76 for moderate drinking and OR 0.34, 95% CI 0.16–0.74 for heavy drinking. Similar estimates for males	
		(25)	
		Relation between alcohol consumption (never/former vs. current) and severe periodontitis (presence/absence): OR: 1.45, 95% CI 0.82–2.57 (non-significant)	
		(27)	
		Various follow-up periods and drinking information were analyzed. In the most reasonable analysis (5 years' follow-up time with high respondence to follow-up), heavy alcohol consumption (>7 units per week for women and >14 units per week for men) vs. light consumption (0–3 units per week for women and 0–7 units per week for men) showed an OR of 4.64, 95% CI 1.1; 19.42 for periodontitis	
		(28)	
		Frequent and infrequent alcohol consumption (daily/weekly intake vs. no/annual/monthly intake) and every tooth loss: Prevalence OR 1.54, 95% CI 1.20–1.56	
		(30)	
		Heavy alcohol consumption (≥40 g for men, ≥20 g for women vs. non-drinking) and risk of periodontal disease (assessed by clinical attachment level, CAL): OR: 2.44, 95% CI 1.03–5.78	
		(31)	
Coffee	Three studies	Number of teeth within four categories of increasing coffee consumption (0 cups/day, 1–2 cups/day, 3–6 cups/day, ≥7 cups/day). Less teeth were observed with increasing coffee intake. ANOVA of lowest (0 cups/day) and highest (≥7 cups/day) category was significant *p* <0.001	⊕ Low
	*n* = 1835	(22)	
		Logistic regression analysis between coffee consumption (≥1 cup/day vs. <1 cup/day) and risk of severe periodontitis: OR: 0.55, 95% CI 0.32–0.92	
		(27)	
		Regression analysis between coffee consumption (cups/day) and oral microbiota richness: negative regression coefficient of −0.216, 95% CI −1.038 to 0.606, *p* = 0.606	
		Regression analysis between coffee consumption (cups/day) and microbiota diversity: positive regression coefficient of 0.002, 95% CI −0.013 to 0.018, *p* = 0.77	
		Regression analysis between coffee (cups/day) and oral microbiota evenness: positive regression coefficient of 0.001, 95% CI −0.001 to 0.002, *p* = 0.52	
		(32)	
Milk	Two studies	Regression analysis between Milk and Milk Products (MMP) (g/Kg) and periodontal disease events over 6 years: negative regression coefficient of −0.10, 95% CI 0.20–0.07, *p* = 0.035	⊕ Very low
	*n* = 735	(24)	
		Logistic regression analysis between total dairy calcium (> recommended mg/day vs. < recommended mg/day) and risk of periodontitis: OR: 0.76, 95% CI 0.58–0.99, *p* = 0.04	
		Logistic regression analysis between total dairy whey (≥ 9.6 g/day vs. <9.6 g/day) and risk of periodontitis: OR: 0.75, 95% CI 0.58–0.97, *p* = 0·03	
		(26)	
Tea	One study	Regression analysis between tea (cups/day) and oral microbiota richness: positive regression coefficient of 1.473, 95% CI 0.015–2.931, *p* = 0.05	⊕ Very low
	*n* = 938	Regression analysis between tea (cups/day) and microbiota diversity: positive regression coefficient of 0.039, 95% CI 0.011–0.067, *p* = 0.006	
		Regression analysis between tea (cups/day) and oral microbiota evenness: positive regression coefficient of 0.004, 95% CI 0.001–0.007, *p* = 0.002	
		(32)	
Sugary beverages	One study	Logistic regression analysis between sugary beverages consumption (>1drink/day vs. ≤ 1drink/day) and risk of tooth loss: OR: 4.52; *p* = <0.01	⊕ Very low
	*n* = 308	(28)	

*OR, odds ratio; CI, confidence interval; p, p-value; OR, odds ratio; CI, confidence interval*.

## Discussion

The present systematic review aimed to address the conceptual hypothesis of the existence of a link between beverage consumption and oral health in the aging population. To this end, the body of evidence on alcohol intake and beverage consumption as milk, tea, coffee, and sugar-sweetened drinks was examined in relation to poor oral health outcomes, including periodontal disease, oral dysbiosis, and tooth loss. A major result of this systematic review was the lack of good studies filling the gap of knowledge in regard to the research question. Nevertheless, some interesting findings were revealed, such as a potential role of high alcohol intake in the development of periodontal disease and probably tooth loss. Tooth loss could also be associated with the consumption of sugary beverages and coffee, although only one eligible study of each had been retrieved (18, 24). Periodontal disease, on the other hand, seems to be potentially inversely affected by the high consumption of beverages such as milk or coffee. Only one study investigated the role of beverages (tea, coffee) in the microbiota composition of the oral cavity in the elderly. The overall quality of the evidence was rated as moderate for alcohol intake but low to very low for beverages.

Several biological pathways have been proposed to explain the association between alcohol and oral health. These pathways were based on intrinsic features of alcoholic beverages. Alcoholic (carbonated) drinks may lead to a rise in salivary acid levels of the mouth, resulting in a drop in salivary pH and the consequent risk of tooth erosion (33, 34). Among alcoholic beverages, wine is particularly low in pH, and this makes erosions quite common among drinkers (35), whereas beer is carbonated. Furthermore, alcohol can damage the soft tissues of the mouth, leading to periodontal disease. Moreover, alcohol slows down the salivary flow, which might explain why alcohol drinkers experience an increase in dental plaque, and thus a higher risk of both tooth decay and discoloration, and of receding gums (33). The saliva flow helps to neutralize acids released by plaque, which further fights tooth decay. A lack of saliva allows acids to accumulate and cause gum disease and tooth decay, along with periodontal disease (36).

The current review identified sugary drinks and coffee as fostering tooth loss. A recent meta-analysis reporting on the association between sugary beverages and dental caries or erosion found a positive dose-response gradient for caries (11). In general, sugar is a substrate supporting bacterial flora that breaks down enamel and dentin and promotes the development of caries, and this may support biological plausibility of our findings. Due to the cross-sectional design of most of the studies included in this review, no claims about temporality can be made, but the positive dose-response relationship of the aforementioned meta-analysis supports a potential causative link. Moreover, coffee is often sweetened with sugar or syrup, leading to bacterial fermentation and disruption of the enamel surface.

Other effects of beverages on oral health could be related to bone metabolism in general. Caffeine consumption has been reported to be involved in altering calcium metabolism and reducing bone mineral density, possibly due to its ability to inhibit osteoblast development and thus the expression of vitamin D receptors on osteoblast surfaces (37). As such, heavy coffee consumption has been reported to be associated with a higher risk of osteoporosis and osteoporotic fractures (38, 39). The observed small beneficial association of milk regarding periodontal disease in adults is in line with literature considering milk and dairy products, containing micro and macronutrients such as calcium and casein, to be helpful in preserving tooth mineralization and protecting against the early onset of cariogenic bacteria (40). In this regard, a large Danish cross-sectional study supported the hypothesis that higher intakes of calcium, casein, and whey may contribute to a lower risk of periodontitis over a wide age range, although causality cannot be inferred (26). Aligned with these latter points, an earlier observation conducted by Barrett-Connor and colleagues indicated that lifelong caffeinated coffee intake only preserved bone mineral density in regular milk drinkers. As such, it seems that caffeine- or coffee-induced calcium and bone loss may be compensated by adequate calcium intake from milk (41). By contrast, the weak positive role of coffee on periodontal disease is supported by a recent report showing that coffee consumption may be protective against periodontal bone loss in adult males (42). These authors found a small protective association of coffee consumption (≥1 cup/day) with periodontal health, particularly in reducing the number of teeth suffering from alveolar bone loss; they attributed this finding to antioxidant nitrogen compounds, vitamins, minerals, and phenols to be found in coffee.

Data on tea were confined to the report by Peters et al. (32), observing an overall improved richness and composition of the oral microbiota in tea drinkers. This observation is in line with other findings suggesting a beneficial role of green tea on oral bacterial networks estimated in the stool of healthy subjects (43). Also, considering existing scientific assumptions about caries and periodontal disease conditions as being associated with reduced oral microbiome diversity (44), this would suggest a potential pathway to good oral health driven by tea consumption.

In the present study, the limited data and the heterogeneity of exposure variables in relation to oral health outcomes lower the reliability in quantitative terms of this narrative and qualitative meta-analysis. Some other limitations should also be taken into account. Firstly, the designs were different across the selected studies, the cross-sectional design being more common, thus leaving little space for discussion of a causal inference. The statistical method of elucidating effects (association) of alcohol and different beverages consumption on oral health, even if the definition was the same, was different across the studies in terms of both the assessment tools used and of the specific description of the beverage type. Especially concerning alcohol, the exposure of interest was the amount as such, rather than the type of alcohol beverage consumed. Also, selected studies differed in sample size and number of beverages considered, alcohol intake being the major focus.

The present systematic review highlighted the importance of considering beverages consumption, in particular alcohol, in protocols to preserve oral health and prevent oral frailty during aging. More research into other beverages and oral cavity outcomes is needed, not covered here due to lack of studies in the elderly, as well as more and better studies on the relations addressed here. We see this review as a first step toward a more thorough acquisition of evidence about this research question (20), which is a prerequisite of an informed decision-making process in public health nutrition. Nowadays, the preventive management of geriatric syndromes is becoming increasingly imperative in a multidisciplinary setting.

## Data Availability Statement

The original contributions presented in the study are included in the article/Supplementary Material, further inquiries can be directed to the corresponding author.

## Author Contributions

RZ and FC: conceptualization, research, resource provision, data collection, writing original version, and visualization. HB: review and correction. RZ, VD, and ML: research and data collection. FP, GG, and GD: conceptualization, validation, review, and correction. RS and HB: conceptualization, validation, review and correction, and visualization. All authors contributed to the article and approved the submitted version.

## Funding

This work was supported by Italian Ministry of Health with “Ricerca Corrente 2020” funds.

## Conflict of Interest

The authors declare that the research was conducted in the absence of any commercial or financial relationships that could be construed as a potential conflict of interest.

## Publisher's Note

All claims expressed in this article are solely those of the authors and do not necessarily represent those of their affiliated organizations, or those of the publisher, the editors and the reviewers. Any product that may be evaluated in this article, or claim that may be made by its manufacturer, is not guaranteed or endorsed by the publisher.
