# Supplementary material for: Beverages Consumption and Oral Health in the Aging Population: A Systematic Review
Source: Front Nutr. 2021 Oct 27;8:762383. doi: 10.3389/fnut.2021.762383 (PMC8579113; doi:10.3389/fnut.2021.762383)
Supplement: Supplementary file 1 [file Data_Sheet_1.PDF]

Supplementary Table S1. Search strategy used in the US National Library of Medicine (PubMed), Medical Literature Analysis, and Retrieval System Online (MEDLINE). The descriptors had been adapted to the other databases.

Strategy	Descriptors used
# 1	(milk[tiab]) OR (drink*[tiab]) OR (alcohol*[tiab]) OR (tea*[tiab]) OR (coffee[tiab]) OR (beer[tiab]) OR (wine[tiab]) OR (spirit*[tiab]) OR (whiskey [tiab]) OR (rum[tiab]) OR (gin[tiab]) OR (gin[tiab]) OR (vodka[tiab]) OR (beverage*[tiab])
# 2	(periodont*[tiab]) OR (oral microbiota[tiab]) OR (oral microbioma[tiab]) OR (tooth loss[tiab]) OR (number of teeth[tiab]) OR (gingivitis[tiab]) OR (oral health[tiab])
# 3	(review[tiab]) OR (narrative review[tiab]) OR (systematic review[tiab]) OR (editorial[tiab]) OR (perspective[tiab]) OR (letter[tiab]) OR (commentary[tiab])
# 5	(#1 AND #2) NOT #3
Number of papers:1308 Date: 31 May 2021. Age 60+	
