# Supplementary material for: Beverages Consumption and Oral Health in the Aging Population: A Systematic Review
Source: Front Nutr. 2021 Oct 27;8:762383. doi: 10.3389/fnut.2021.762383 (PMC8579113; doi:10.3389/fnut.2021.762383)
Supplement: Supplementary file 2 [file Data_Sheet_2.pdf]

(Panel A)

	Study question	Population	Participation rate	Inclusion criteria	Sample size	Exposure prior to outcome	Sufficient time frame	Different levels of exposure	Exposure measures	Multiple exposure	Outcome measures	Blinding of outcome	Loss to follow-up	Confounding
Drake C.W. et al., 1995	✓	✓	✓	✓	✗	✓	✓	✓	✓	✗	✗	✗	✗	✗
Norlén P. et al., 1996	✓	✓	✓	✓	✗	-	-	✓	✓	-	✓	✗	-	✗
Hanioka T. et al., 2007	✓	✓	-	-	✗	-	-	✓	✓	-	✓	✗	-	✓
Yoshihara A. et al., 2009	✓	✓	✓	✓	✗	✓	✓	✓	✓	✓	✓	✗	-	✓
Heegaard K. et al., 2011	✓	✓	✗	✓	✗	-	-	✓	✓	-	✗	✗	-	✓
Adegboye A.R.A. et al., 2012	✓	✓	✗	✓	✗	-	-	✓	✓	-	✓	✗	-	✓
Machida T. et al., 2014	✓	✓	✓	✓	✗	-	-	✓	✓	-	✓	✗	-	✓
Hach M. et al., 2015	✓	✓	✗	✓	✗	✓	✓	✗	✓	✓	✓	✗	-	✓
Tiwari T. et al., 2016	✓	✓	✓	✓	✗	-	-	✗	✓	-	✗	✗	-	✓
Laguzzi P.N. et al., 2016	✓	✓	✓	✓	✗	-	-	✓	✓	-	✓	✗	-	✓
Suwama K et al., 2018	✓	✓	✓	✓	✗	-	-	✓	✓	-	✓	✗	-	✗
Peters B.A. et al., 2018	✓	✓	✓	✓	✗	✗	-	✓	✓	✗	✓	✗	✓	✓

✓Reported: element reported appropriately in study
x Not reported: element not mentioned in study
- Not applicable: due to study design, element not applicable to report
