# Supplementary figures and images for: Beverages Consumption and Oral Health in the Aging Population: A Systematic Review
Source: Front Nutr. 2021 Oct 27;8:762383. doi: 10.3389/fnut.2021.762383 (PMC8579113; doi:10.3389/fnut.2021.762383)

**Panel B**

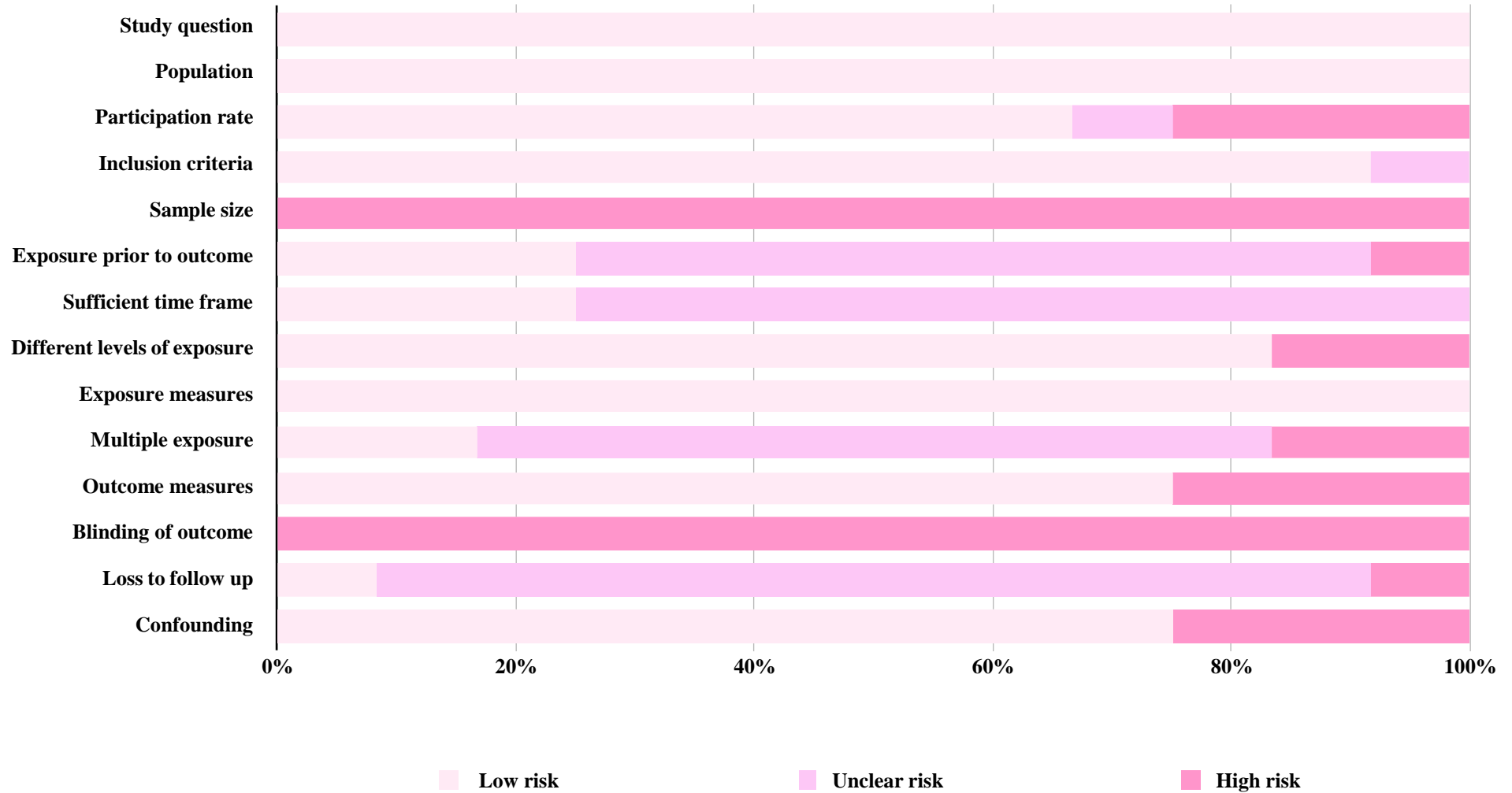

Supplement: Supplementary file 3 [file Data_Sheet_3.pdf]
